# Ultrasound-guided para-umbilical block: a pediatric case

**DOI:** 10.1186/s40981-017-0105-6

**Published:** 2017-06-07

**Authors:** Masayuki Akatsuka, Takeshi Murouchi, Johji Arakawa, Michiaki Yamakage

**Affiliations:** 1 Department of Anesthesia, Japanese Red Cross Kitami Hospital, North6 East2, Kitami, Hokkaido 090-0026 Japan; 2 Department of Anesthesiology, Sapporo Medical University School of Medicine, West16 South1, Chuo-ku, Sapporo, Hokkaido 060-8543 Japan

**Keywords:** Para-umbilical block, Perioperative analgesia, Pediatric anesthesia

## Abstract

**Background:**

Rectus sheath block is a common peripheral nerve block for patients undergoing umbilical hernia repair surgery. However, rectus sheath block alone can affect only anterior branches of intercostal nerves and, therefore, is incomplete for postoperative analgesia for the anterior abdomen, which is innervated by both anterior and lateral branches. We describe a successful perioperative analgesia with ultrasound-guided para-umbilical block after pediatric umbilical hernia surgery.

**Case presentation:**

A 4-year-old child underwent hernia repair surgery. Following induction of general anesthesia, the anatomy of the umbilical region was observed under ultrasound with a 5–10-MHz linear probe. An ultrasound-guided injection between the rectus abdominis muscle and the posterior lobe of the rectus sheath and an injection into the subcutaneous space around the umbilicus were performed. The peripheral nerve block was effective during surgery, and the patient required no additional rescue analgesia during the perioperative period. There were no complications.

**Conclusion:**

We performed ultrasound-guided para-umbilical block with four injections. This peripheral nerve block could be an efficient technique for complete perioperative analgesia.

## Background

Umbilical hernia repair is a common surgical procedure in pediatric surgery. It is usually carried out in children over 2 years old under general anesthesia, sometimes combined with regional blocks. Rectus sheath block has been long used for this purpose [1]. However, rectus sheath block can affect only anterior branches of intercostal nerves, and the effect could be incomplete; anterior abdominal regions are innervated by both anterior and lateral branches. Although several anatomical studies have shown many variations in the innervation of the umbilical region [2–4], there have been few studies on postoperative pain control in children after umbilical surgery with umbilical nerve block. Here, we describe a successful perioperative pain management of a pediatric umbilical hernia surgery with ultrasound-guided para-umbilical nerve block.

## Case presentation

This case report was approved by our institutional research ethics committee, and the permission of publication from the parents of the patient was waived.

An otherwise healthy 4-year-old child (104 cm/15 kg) with umbilical hernia was planned to undergo hernia repair surgery. General anesthesia combined with regional anesthesia was planned.

**Fig. 1:**
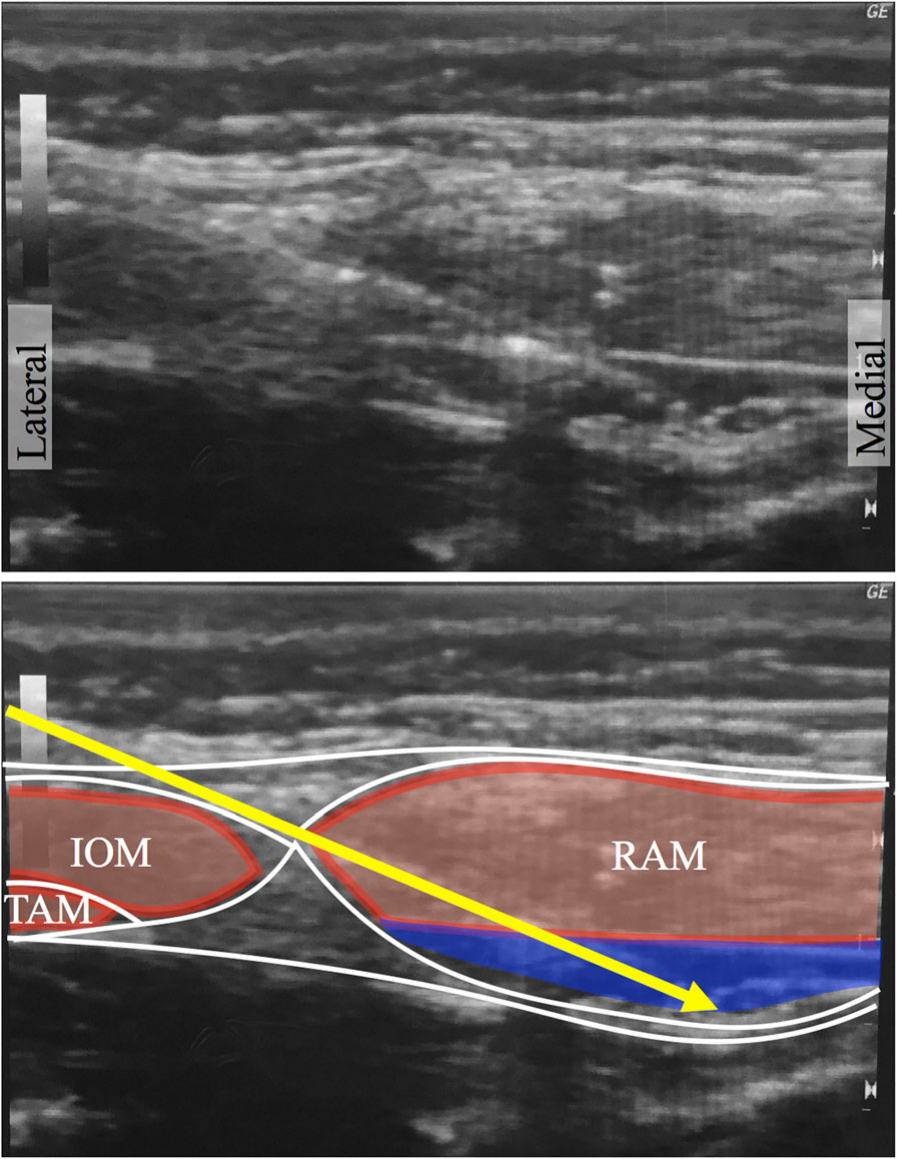
The injection between the rectus muscle and the posterior sheath. The tip of the needle (*yellow arrow*) is on the posterior lobe of the rectus sheath. The deposition of injected local anesthetic (*blue semicircle*) is observed behind the rectus abdominis muscle. *RAM* rectus abdominis muscle, *IOM* internal oblique muscle, *TAM* transversus abdominis muscle

**Fig. 2:**
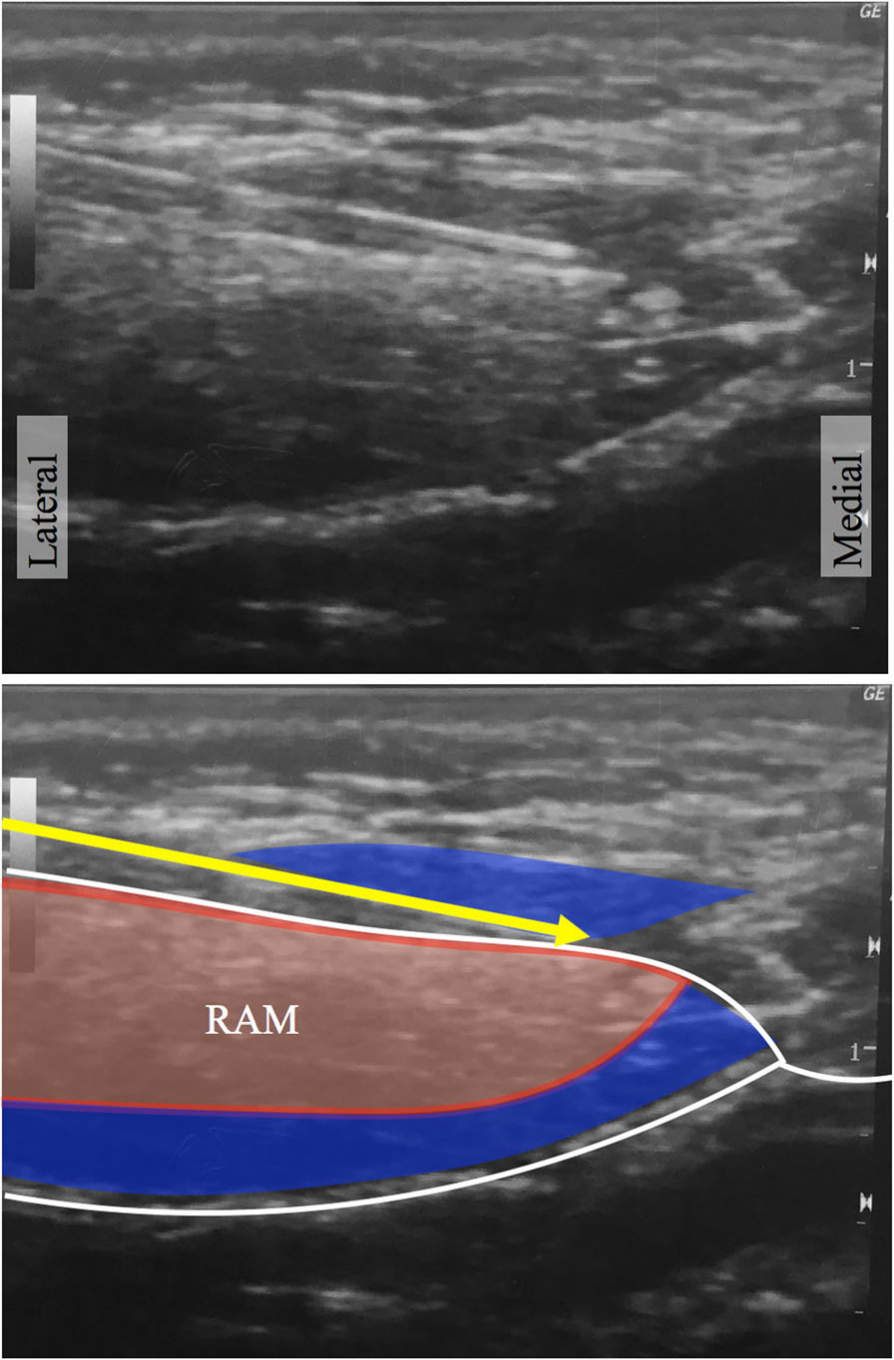
The injection in the subcutaneous tissue. The tip of the needle (*yellow arrow*) is in the subcutaneous space around the umbilicus. The depositions of injected local anesthetic (*blue*) are also observed inside the rectus sheath. *RAM* rectus abdominis muscle

Premedication was not given in the case. Standard monitors were attached to the patient. Anesthesia was induced with 5% sevoflurane via facemask, and the venous access was established after general anesthesia. The patient was intubated after muscle relaxation had been acquired with 1.0 mg/kg of rocuronium. Anesthesia was maintained with 2–2.5% sevoflurane, and mechanical ventilation by pressure control was maintained throughout the surgery. The ultrasonographic anatomy of the umbilical region was observed using a 5–10-MHz linear probe. The ultrasound probe was placed on the anterior abdomen in the transverse plane beside the umbilicus. The lateral edge of rectus abdominis muscle (RAM) was localized, and internal oblique muscle (IOM) was identified. A 22G short-beveled needle was introduced in-plane, with the probe on the lateral edge of the RAM. The needle tip was advanced until it was placed between the RAM and the posterior lobe of the rectus sheath. The needle position was confirmed with a small amount of saline; then, 4 mL of 0.25% ropivacaine was administered under real-time ultrasound guidance (Fig. 1). Then, the needle tip was pulled back to the subcutaneous area and advanced in-plane with the probe to the subcutaneous space around the umbilicus. A subcutaneous fan-shaped injection of 4 mL of 0.25% ropivacaine was performed (Fig. 2). Then, the same maneuvers were performed on the contralateral side.

Surgery was performed without any changes in heart rate and blood pressure. No additional rescue analgesia was therefore given in the operating room. The acute pain control was complete after emergence.

The postoperative course was uneventful, and there was no episode of nausea or vomiting. There was no additional rescue analgesia during the entire postoperative period, and the case scored 4 in the Children’s Hospital Eastern Ontario Pain Scale (CHEOPS) [5] after surgery and had not scored ≥5 until hospital discharge.

### Discussion

In this case report, para-umbilical block [6] was performed for complete analgesia in umbilical hernia repair surgery under real-time ultrasound guidance. The result of this case suggests that ultrasound-guided para-umbilical block is effective as both anesthesia and postoperative analgesia, theoretically affecting both the anterior and lateral branches of intercostal nerves innervating the umbilical region.

A previous study showed that a landmark-based para-umbilical block provided both intraoperative analgesia and postoperative analgesia in children undergoing umbilical hernia repair [6]. The authors suggested that the injections over the rectus sheath and inside the rectus sheath can improve capture of aberrant anterior cutaneous branches. Another study showed that an ultrasound-guided peripheral block of the bilateral 10th intercostal nerves in the lateral edge of RAM was effective [7]. Both the approach [7] and rectus sheath block cannot affect aberrant anterior branches nor lateral cutaneous branches. Therefore, we performed ultrasound-guided para-umbilical block to administer the local anesthetic on the posterior lobe of the rectus sheath and in subcutaneous tissue around the umbilicus in a fan-shaped fashion. The spread of the local anesthetic on the sheath and subcutaneous tissue was clearly observed.

The main advantage of this block is completeness. This case needed no rescue during the perioperative period. The patient had no postoperative pain so that she was able to jump for fun and run 3 h after returning to the ward without pain. She had no complications such as hematoma and peritoneal puncture after the regional technique.

It is only one case we suggested the efficient nerve block for analgesia during the perioperative period. Prospective trials of the ultrasound-guided para-umbilical block are warranted to elucidate characteristics of patients that may affect the success of the para-umbilical block.

## Conclusions

We performed para-umbilical block with four injections under ultrasound guidance. This approach might provide complete perioperative analgesia after umbilical hernia repair.

## Abbreviations

RAM: Rectus abdominis muscle
IOM: Internal oblique muscle
TAM: Transversus abdominis muscle
CHEOPS: Children’s Hospital Eastern Ontario Pain Scale
